# Prediction performance of linear models and gradient boosting machine on complex phenotypes in outbred mice

**DOI:** 10.1093/g3journal/jkac039

**Published:** 2022-02-15

**Authors:** Bruno C Perez, Marco C A M Bink, Karen L Svenson, Gary A Churchill, Mario P L Calus

**Affiliations:** 1 Hendrix Genetics B.V., Research and Technology Center (RTC), 5830 AC Boxmeer, The Netherlands; 2 The Jackson Laboratory, Bar Harbor, ME 04609, USA; 3 Wageningen University & Research, Animal Breeding and Genomics, 6700 AH Wageningen, The Netherlands

**Keywords:** Genomic Prediction, GenPred, Shared Data Resources

## Abstract

We compared the performance of linear (GBLUP, BayesB, and elastic net) methods to a nonparametric tree-based ensemble (gradient boosting machine) method for genomic prediction of complex traits in mice. The dataset used contained genotypes for 50,112 SNP markers and phenotypes for 835 animals from 6 generations. Traits analyzed were bone mineral density, body weight at 10, 15, and 20 weeks, fat percentage, circulating cholesterol, glucose, insulin, triglycerides, and urine creatinine. The youngest generation was used as a validation subset, and predictions were based on all older generations. Model performance was evaluated by comparing predictions for animals in the validation subset against their adjusted phenotypes. Linear models outperformed gradient boosting machine for 7 out of 10 traits. For bone mineral density, cholesterol, and glucose, the gradient boosting machine model showed better prediction accuracy and lower relative root mean squared error than the linear models. Interestingly, for these 3 traits, there is evidence of a relevant portion of phenotypic variance being explained by epistatic effects. Using a subset of top markers selected from a gradient boosting machine model helped for some of the traits to improve the accuracy of prediction when these were fitted into linear and gradient boosting machine models. Our results indicate that gradient boosting machine is more strongly affected by data size and decreased connectedness between reference and validation sets than the linear models. Although the linear models outperformed gradient boosting machine for the polygenic traits, our results suggest that gradient boosting machine is a competitive method to predict complex traits with assumed epistatic effects.

## Introduction

The use of genome-wide markers as predictor variables for individuals’ unobserved phenotypes (Meuwissen et al. 2001) based on a reference population is known as genomic prediction (GP). In the past decade, high-throughput genotyping technologies made GP accessible and facilitated large-scale use of GP for animal (Boichard et al. 2016) and plant breeding (Bhat et al. 2016), and in human genetics (Lappalainen et al. 2019). For animals and plants, GP has reduced breeding costs and speeded up breeding programs as individuals of interest can be selected in earlier stages of life, while reducing costs for performance testing. In humans, major efforts have been put into developing GP to score disease risks (Duncan et al. 2019), aiming for more personalized medicine in the future (Barrera-Saldaña 2020).

Currently, most GP models assume that observed phenotypes are controlled by numerous loci with additive effects throughout the genome and this approach has provided a robust performance in most cases (Meuwissen et al. 2001; Calus 2010). However, in the literature, it has been suggested that the genetic architecture of complex traits may involve significant proportions of nonadditive genetic (dominance or epistasis) effects (Mackay 2014) and that these could be much more common than previously thought (Sackton and Hartl 2016). Although accounting for nonadditive effects into parametric GP models has been reported to improve predictive performance (Forsberg et al. 2017) of phenotypes, implementing variable selection to prioritize among all possible SNP by SNP interactions is computationally too costly for any practical application.

Machine learning (ML) has been successfully used in many fields for text, image, and audio processing at huge data volumes. Recently, these algorithms have found many applications in GP for offering an opportunity to model complex trait architectures in a much simpler framework than parametric models (Nayeri et al. 2019; Montesinos-López et al. 2021; van Dijk et al. 2021). ML algorithms are free from model specification, can accommodate interactions between predictive variables, and deal with large numbers of predictor variables by performing automatic variable selection (Jiang et al. 2009; Li et al. 2018).

Howard et al. (2014), Ghafouri-Kesbi et al. (2017), and Abdollahi-Arpanahi et al. (2020) have compared the predictive performance of linear and ML models for simulated phenotypes controlled by additive or nonadditive effects. In general, linear models were able to outperform ML models for traits controlled by additive effects; however, they failed to do so when used to predict traits with purely epistatic architecture. The superiority of ML over traditional linear models was most notable for traits controlled by a small number of loci (100) with nonadditive effects. For this type of scenario, Ghafouri-Kesbi et al. (2017) and Abdollahi-Arpanahi et al. (2020) also showed a consistent good performance of the gradient boosting machine (GBM) algorithm (Friedman 2001), which has previously been reported to provide robust predictive ability when compared to other methods in the context of GP (Gonzalez-Recio and Forni 2011; Jiménez-Montero et al. 2013; González-Recio et al. 2013, 2014; Grinberg et al. 2020; Srivastava et al. 2021).

Although results in simulated data suggest the superiority of ML models in the presence of epistatic effects, the performance of such models has been less consistent for GP using real data. Zingaretti et al. (2020) observed that convolutional neural networks (CNN) had 20% higher predictive accuracy than linear models for GP of a trait with a strong dominance component (percentage of culled fruit) in strawberry but underperformed for traits with predominant additive effects. On the other hand, in Azodi et al. (2019), ML did not consistently outperform linear models for traits with strong evidence of underlying nonadditive architectures (for example height in maize and rice). The authors describe that ML models presented less stable prediction across traits than linear models. Similar results were reported by Bellot et al. (2018) while investigating the performance of GP for several complex human phenotypes. An important aspect to consider when investigating performance of GP models is that for most livestock and plant species there is currently limited knowledge of the genetic architecture of economically important traits. This makes it difficult to perform inference about the real reasons why ML outperforms linear models in specific situations. This could be overcome by considering data from populations for which knowledge of the genetic architecture of traits is more extensively and accurately described.

The diversity outbred (DO) mice population is derived from 8 inbred founder strains (Svenson et al. 2012). It is an interesting resource for high-resolution genetic mapping by having a low level of genetic relationship between individuals, low extent of LD (Churchill et al. 2012) and uniformly distributed variation across genomic regions of known genes (Yang et al. 2011). This structure represents an advantage over classical inbred strains of mice or livestock populations, which have limited genetic diversity (Yang et al. 2011). These aspects allow the investigation of relevant traits in a structured scheme that closely reflects the genetic mechanisms of human disease (Churchill et al. 2012; Svenson et al. 2012).

In the present study, the objective was to compare the performance of GBM to several linear models [GBLUP, BayesB, and elastic net (ENET)] for predicting 10 complex phenotypes in the DO mice population. All models were applied for scenarios where data were not available for 1 or more generations in between the reference and validation sets. In addition, we explore the use of feature selection from the GBM algorithm as a tool for subsetting relevant markers and to improve prediction accuracy through dimensional reduction.

## Materials and Methods

### Data

#### Phenotypes

**Fig. 1.**
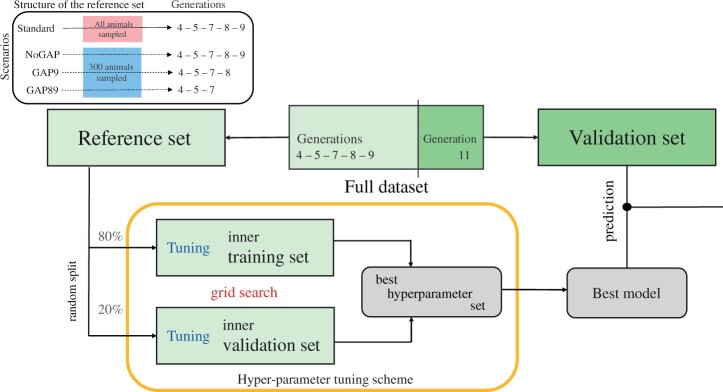
Graphical representation of the cross-validation scheme and hyper-parameter tuning grid-search scheme implemented to obtain the best GBM and ENET models. In addition, the reference subset structure for scenarios is presented (top left).

The DO mice data comprising 835 animals were obtained from The Jackson Laboratory (Bar Harbor, ME). The animals originated from 6 nonoverlapping generations (4, 5, 7, 8, 9, and 11) in which males and females were represented equally. The total number of animals per generation was 97, 48, 200, 184, 99, and 197 for generations 4, 5, 7, 8, 9, and 11, respectively, but numbers of missing records varied across traits (Fig. 1). The mice were maintained on either standard high fiber (chow, *n* = 446) or high fat and sugar diet (*n* = 389) from weaning until 23 weeks of age. The proportion of males and females within each diet category was close to 50–50 for all generations. The same was observed for the frequency of males and females within each litter-generation combination (2 litters per generation). A detailed description of husbandry and phenotyping methods can be found in Svenson et al. (2012).

Table 1 shows a comprehensive description of each trait regarding dataset size, estimated heritability (using the dataset available) and assumed genetic architecture with associated literature. To assess the evidence of nonadditive effects involved in the analyzed traits, we have also estimated variance components fitting both the additive and additive-by-additive (Vitezica et al. 2017) genomic relationship matrices. For all traits, we considered the full dataset (combining reference and validation subset) to perform this task. Results from these analyzes are presented as Supplementary Table 1. Among all phenotypes available, we chose 10 traits based on their distinct assumed genetic architectures from previous results with the same dataset (Li and Churchill 2010; Churchill et al. 2012; Zhang et al. 2012; Tyler et al. 2016, 2017; Keller et al. 2019; Keenan et al. 2021) and other populations (Chitre et al. 2020). The analyzed traits were bone mineral density at 12 weeks (BMD), body weight at 10, 15, and 20 weeks (BW10, BW15, and BW20, respectively), circulating cholesterol at 19 weeks (CHOL), adjusted body fat percentage at 12 weeks (FATP), circulating glucose at 19 weeks (GLUC), circulating triglycerides at 19 weeks (TRGL), circulating insulin at 8 weeks (INSUL), and urine creatinine at 20 weeks (UCRT). These traits can be categorized into measurements of body composition (weights and fat percentage), clinical plasma chemistries (triglycerides, glucose, insulin), and urine chemistry (urine creatinine).

**Table 1.** Number of available observations (*N*), estimated heritability, assumptions from literature regarding the genetic architecture of the trait and references.

Trait	*N*	Heritability^a^	Genetic architecture	Reference
BMD	831	0.36	Evidence of epistatic effects	Tyler et al. (2016)
BW10	834	0.42	Highly polygenic	Tyler et al. (2017)
				Chitre et al. (2020)
BW15	829	0.39	Highly polygenic	Tyler et al. (2017)
				Chitre et al. (2020)
BW20	827	0.38	Highly polygenic	Tyler et al. (2017)
				Chitre et al. (2020)
FATP	831	0.37	Highly polygenic	Tyler et al. (2017)
CHOL	819	0.29	QTL with high effect	Stewart et al. (2010)
			Evidence of epistatic effects	Li and Churchill (2010)
				Zhang et al. (2012)
GLUC	816	0.18	Evidence of epistatic effects	Stewart et al. (2010)
				Chen et al. (2017)
INSUL	820	0.30	QTL with high effect	Keller et al. (2019)
TRGL	820	0.22	Highly polygenic	Stewart et al. (2010)
UCRT	799	0.21	Highly polygenic	Perry (2019)
			Evidence of dominance effects	Zhang et al. (2012)

a Standard error was close to 0.08 for all traits.

Prior to any analyses performed in this study, phenotypic records were precorrected for fixed effects of diet, generation, litter, and sex. The precorrected phenotype ($y^{*})$ can be represented by:

$$y^{*}=a+e$$

where a is the vector of animal additive genetic effects and e the vector of residuals.

#### Genotypes

Mice from 8 distinct founder strains were genotyped using either the MUGA or MegaMUGA SNP arrays (Morgan et al. 2015). The variant calls from the arrays in the animals contained in the current dataset were converted to founder haplotypes using a hidden Markov model (Gatti et al. 2014), which uses the SNP genotypes in an individual mouse to infer transition points between different DO founder haplotypes. After that, the probability of each parental haplotype at each SNP position in the genome (Gatti et al. 2014) was used to derive SNP genotype probabilities. This effectively fills missing genotype calls and provides error correction by locally smoothing the SNP calls to be consistent with the haplotype structure of the mosaic DO mouse genome. To accomplish this, we used functions available in the “QTL2” R package (Broman et al. 2019).

The complete genotype file used for the analyses was composed of 60,640 markers reconstructed from the diplotype probabilities from the MUGA and MegaMUGA on an evenly spaced grid, and the average distance between markers was 0.0238 cM. The full genotype data were cleaned based on the following criteria. Variants were removed if they had a minor allele frequency of <0.05, a call rate of <0.90, or a linear correlation with a subsequent SNP of >0.80 (one of the pair was randomly removed). Animals were removed if they had a call rate <0.90. After quality control, a total of 50,112 biallelic SNP markers were available for the mice with both phenotypic and genotypic records. We included as supplementary material a description of the number of SNP retained per chromosome after quality control (Supplementary Table 2) and the linkage disequilibrium (measured by *r*^2^) extent in the DO mouse genotype data (Supplementary Fig. 1).

### GP models

#### GBLUP

The statistical model of GBLUP is:

$$y^{*}=1\mu +a+e$$

where $y^{*}$ is the vector of precorrected phenotypes, **1** is a vector of ones, μ is the intercept, a is the vector of random additive genetic values, where $a\;∼\;N(0,\;G\sigma_{a}^{2})$, and G is the additive genomic relationship matrix between genotyped individuals. It is constructed following the second method described by VanRaden (2008) as $\frac{{\mathrm{ZZ}}^{'}}{m}$ where Z is the matrix of centered and standardized genotypes for all individuals and m is the number of markers, and $\sigma_{a}^{2}$ is the additive genomic variance, e is the vector of random residual effects where $e\;∼\;N(0,\;{I\sigma }_{e}^{2})$ with $\sigma_{e}^{2}$ being the residual variance, and I is an identity matrix. As mentioned before, we used this method to estimate genetic parameters for all analyzed traits using additive and additive-by-additive genomic relationship matrices. GBLUP was implemented using a Bayesian approach using the BGLR package (Pérez and de los Campos 2014). The Gibbs sampler was run for 150,000 iterations, with a 50,000 burn-in period and a thinning interval of 10 iterations. Consequently, inference was based on 10,000 posterior samples.

#### BayesB

BayesB has been widely used for GP (Meuwissen et al. 2001). It provides a linear model with variable selection ability. The phenotype of the *i*th individual is expressed as a linear regression on markers:

$$y^{*}=1\mu +Z\beta +e$$

where $y^{*}$ is the vector of precorrected phenotypes, **1** is a vector of ones, μ is the intercept, β is the vector of random effect of markers, Z is the incidence matrix for markers, e is a random residual where $e\;∼\;N(0,\;{I\sigma }_{e}^{2})$ with $\sigma_{e}^{2}$ being the residual variance, and I is an identity matrix. In contrast to GBLUP, BayesB assumes a priori that not all markers contribute to genetic variation of given trait. For BayesB, all markers are assumed to have a 2-component mixture prior distribution. Any given marker has either a null effect with known prior probability, π, or a t prior distribution with probability (1-π), with ν degrees of freedom and scale parameter $s^{2}$. Therefore, marker effects $\beta_{k}\;∼\;N(0,\;\sigma_{gk}^{2})$, where $\sigma_{gk}^{2}$ is the variance of the *k*th SNP effect. The BayesB model was implemented using the BGLR package (Pérez and de los Campos 2014). The Gibbs sampler was run for 120,000 iterations, with a 20,000 burn-in period and a thinning interval of 100 iterations. Consequently, inference was performed based on 10,000 posterior samples.

#### ENET

The ENET is an extension of the Lasso (Friedman et al. 2010) and is considered a robust method in the presence of strong collinearity among predictors, as is the case for genotype data. It can be described by the regression model:

$$y^{*}=1\mu +Z\beta +e$$

where $y^{*}$ is the vector of precorrected phenotypes, β is the vector of random effect of markers, Z is the incidence matrix for markers, e is a random residual where $e\;∼\;N(0,\;{I\sigma }_{e}^{2})$ with $\sigma_{e}^{2}$ being the residual variance, and I is an identity matrix.

ENET uses a mixture of the $\ell_{1}$ (Lasso) and $\ell_{2}$ (ridge regression) penalties and the estimator ${\hat{\beta }}_{\mathrm{ENET}}$ can be formulated as:

$${\hat{\beta }}_{\mathrm{ENET}}=\left(1+\;\frac{\lambda_{2}}{n}\right)\left\{{\mathrm{argmin}}_{\beta }\;{\left\|y-X\beta \right\|}_{2}^{2}+\;\lambda_{2}\;{\left\|\beta \right\|}_{2}^{2}+\;\lambda_{1}{\left\|\beta \right\|}_{1}\right\}$$

where ${\left\|\beta \right\|}_{1}=\sum_{j=1}^{p}\left|\beta_{j}\right|$ is the $\ell_{1}$-norm penalty on β, ${\left\|\beta \right\|}_{2}^{2}=\sum_{j=1}^{p}\beta_{j}^{2}$ is the $\ell_{2}$-norm penalty on β, ${\left\|y-X\beta \right\|}_{2}^{2}=\;\sum_{i=1}^{n}{\left(y_{i}-\;x_{i}^{T}\beta \right)}^{2}$ is the $\ell_{2}$-norm (quadratic) loss function (residual sum of squares), $x_{i}^{T}$ is the *i*th row of X, $\lambda_{1}$ is the parameter that controls the extent of variable selection, and $\lambda_{2}$ is the parameter that regulates the strength of linear shrinkage.

When setting $\alpha =\;\frac{\lambda_{2}}{\left(\lambda_{1}\;+\;\lambda_{2}\right)},$ the ENET estimator is equivalent to the minimizer of:

$${\hat{\beta }}_{\mathrm{ENET}2}={\mathrm{argmin}}_{\beta }\;{\left\|y-X\beta \right\|}_{2}^{2},\;\mathrm{subject}\;\mathrm{to}\;P_{\alpha }\left(\beta \right)=\left(1-\;\alpha \right){\left\|\beta \right\|}_{1}+\alpha {\left\|\beta \right\|}_{2}^{2}\;\leq s\;\mathrm{for}\;\mathrm{some}\;s$$

where $P_{\alpha }\left(\beta \right)$ is the ENET penalty (Zou and Hastie 2005). The ENET is equivalent to ridge regression (Hoerl and Kennard 1970) when α=1, and to the Lasso when α=0. In practice, the $\ell_{1}$ component performs automatic variable selection while the $\ell_{2}$ component ensures that a group of highly correlated variables get effect estimates of similar magnitude.

We implemented the ENET model using the h2o.ai R package (Click et al. 2016). To establish the best hyperparameter set for ENET, we performed a cross-validation (splitting the reference set into 80–20 for train/test sets, as depicted in Fig. 1) on a 2-step scheme. First a grid search of values for the parameter α considering from 0 to 1, in intervals of 0.05. For each value of α, the best value of λ was obtained by computing models sequentially, starting with λ=1 and decreasing it exponentially until 0.01 in up to 20 steps. For each analysis, the best ENET model was chosen by the combination of α and λ parameters obtained from the grid search that yielded the lowest mean squared error of prediction in the test set, and this model was used to predict the validation animals (Supplementary Table 3).

#### GBM

GBM is a flexible ensemble learning technique that combines gradient-based optimization and boosting techniques. Gradient-based optimization uses gradient computations to minimize a model’s loss function in terms of the training data, while boosting applies an iterative process of assembling “weak learners” to obtain a robust predictive machine well suited for regression and classification tasks (Hastie et al. 2009). The algorithm also does automatic feature selection, prioritizing important variables and discarding ones containing irrelevant or redundant information.

As an ensemble method, the gradient boosting method can be expressed as a linear combination of a collection of models:

$$y=\;1\mu +\;\vartheta_{1}h_{1}\left(y;X\right)+\;\vartheta_{2}h_{2}\left(y;X\right)+\;\vartheta_{3}h_{3}\left(y;X\right)+\;\vartheta_{m}h_{m}\left(y;X\right)+\cdots +\;\vartheta_{M}h_{M}\left(y;X\right)+e,$$

where y is the vector of observations, μ is the intercept, $h_{m}\left(y;X\right)(m\;\in \left\{1,\ldots M\right\})$ represents each model applied, $\vartheta_{m}(m\;\in \left\{1,\ldots M\right\})$ is a weight parameter applied to each model, and e is the vector of residuals. In the scope of the present study, it can be described as follows:

$$y^{*}=1\mu +\;\sum_{m=1}^{M}{\vartheta h}_{m}(y^{*};X)+e$$

where $y^{*}$ is the vector of precorrected phenotypes, X is the matrix of genotypes, and all other parameters are as described above. When the algorithm starts, the first model is fitted on the residuals of an initialized prediction based on the distribution of the response variable. From this point, the algorithm fits subsequent models on residuals of the previous model and, at this point, residuals from a model m can be considered residual estimates ($\hat{e}$), in which $\hat{e}\;∼\;N(0,\;\sigma_{e_{m}}^{2})$ and $\sigma_{e_{m}}^{2}$ is the residual variance for model m. Every subsequent model aims to minimize the prediction error from the previous one; therefore, *M* is obtained when no further improvement can be achieved for a given number of iterations. Different parameters can be used to measure that “improvement,” in the present study we used the root mean squared error. The ϑ parameter is used to control how much variance is subtracted from residuals at each iteration, creating a trade-off between number of models and relevance of the SNP. In practice, smaller values of ϑ require assembling more models to reach the same error rate in training data but typically result in better generalization and predictive performance on validation data.

Once the *M* models are assembled, predictions for the validation set may be calculated as:

$$\hat{y}=1\hat{\mu }+\;\sum_{m=1}^{M}{\vartheta \hat{h}}_{m}(X_{i}).$$

More details on the gradient boosting algorithm can be found in Friedman (2001) and Hastie et al. (2009), while the implementation for GP is illustrated in more detail by González-Recio et al. (2010, 2013).

The performance of ML methods can be sensitive to hyperparameters (Azodi et al. 2019). To obtain the best possible results from the GBM algorithm, a grid search approach was used to determine the combination of hyperparameters that maximized prediction performance for each trait. Hyperparameters (and range of values) included were number of trees (*ntree *=* *100, 150, 200, 300, 500, 1,000, 2,000, and 5,000), learning rate (*lrn_rate *=* *0.01, 0.05, and 0.10), and maximum tree depth (*max_depth *=* *2, 3, 5, and 10). For each trait analyzed, the hyperparameter tuning scheme was performed inside the reference subset (cf. ENET and Fig. 1). The best set of hyperparameters was chosen based on the lowest mean squared error obtained from the grid search. Results reported in the present study for GBM model refer to the best performing model out of the grid search for each trait (Supplementary Table 3). We implemented the GBM model using the h2o.ai R package (Click et al. 2016).

### Model performance

Performance of predictions from the models was measured by the accuracy, computed as the Pearson correlation ($r_{y^{*},\hat{y}}$), and the root relative mean squared error (RRMSE) of prediction between predicted values ($\hat{y})$ and precorrected phenotypes ${(y}^{*})$: RRMSE = $\sqrt{\frac{1}{n}\;\sum_{i=1}^{n}({y^{*}-\hat{y})}^{2}}/\sigma_{p}$, where $\sigma_{p}$ is the trait’s phenotypic standard deviation. As described in Fig. 1, we used a forward prediction validation scheme in which animals from older generations (4, 5, 7, 8, and 9) were used as the reference and animals from the younger generation (11) as the validation subset. Uncertainties around the $r_{y^{*},\hat{y}}$ estimates were obtained by using bootstrapping (Davison and Hinkley 1997), implemented in the “boot” R package (Canty and Ripley 2021).

### Impact of the distance between a fixed-size reference and the validation set

Here, we tested the impact of an increase in distance between the reference and validation sets on the prediction performance of each model. To accomplish that, we considered 3 scenarios using generation 11 as validation as before: using generations 4, 5, 7, 8, and 9 as reference (NoGAP), using generations 4, 5, 7, and 8 as reference and omitting phenotypes from generation 9 (GAP9), and using generations 4, 5, and 7 as reference and omitting phenotypes from generations 8 and 9 (GAP89). Considering the full dataset, there were a total of 638 animals from generations 4 to 9 available to be sampled for the validation subset. To analyze the proposed scenarios, the number of animals sampled for the reference subset was kept the same in all scenarios (*N* = 300), with a constraint on the number of animals sampled from each generation to match its representativeness in NoGAP scenario (Supplementary Table 4 for details). The fixed sample size of 300 was arbitrarily chosen based on the number of records available in GAP89, the scenario with the least available data to be sampled for the reference subset (*N* = 345). Every scenario was evaluated in 20 replicates, inference was based on the average and standard deviation of accuracies obtained from replicates. All described models were applied to each of the 20 replicates (in every scenario) considering the same sampled dataset in each replicate across models.

#### Feature importance for dimensionality reduction

For GBM, the importance of a feature is determined by assessing whether that feature was selected to split on during the tree building process, and the contribution of that to decrease the squared error (averaged over all trees) as a result (Friedman and Meulman 2003; Hastie et al. 2009). The feature importance is expressed in a percentage scale that can be ranked to assess the magnitude of importance of each feature.

Here, we investigated if the feature importance scoring performed by the GBM model could be used to preselect markers to be used for GP. The objective was to understand the trade-off between dimensionality reduction and prediction accuracies. To achieve that, for each trait independently we first fitted a GBM model to extract relevant features, i.e. SNPs. In a second step, we considered only a subset of the relevant SNP extracted from the first GBM model into GBLUP, ENET and a second GBM model used for prediction. We did not consider BayesB in this second step, as it does not seem sensible to fit a variable selection method on a limited set of selected variants. After obtaining the importance rank score for SNP in the full panel (50,112 markers), we considered the top 100, 250, 500, and 1,000 features from a GBM model using the cross-validation strategy previously explained as input for GBLUP, ENET, and GBM models. The important features were obtained using the same strategy described for the hyperparameter tuning using a random split (80–20) within the reference subset (Fig. 1).

#### Similarities among top SNPs and prediction rankings

To assess the relationship between model’s prediction at the animal level, we quantified the number of animals in common in the top 20 ranked animals (approximately top 10% of generation 11) from each model. The latter metric gives an indication of the extent to which the same animals would be selected using these different models in a breeding program where each generation 10% of the animals are selected as parents of the next generation. Also, to understand the relationship between predictions from the models at the genome level, we quantified the overlap between the top 1,000 ranked SNP among the models and traits analyzed. For the linear models, SNPs were ranked by their squared estimated effect. In BayesB and ENET, these effects were explicitly estimated from the models while for GBLUP, SNP effects were calculated by back solving from GEBV solutions (Strandén and Garrick 2009). For the GBM model, SNPs were ranked by their importance score (as described above). For any given trait, an “overlapping SNP” between 2 models A and B was defined as any SNP in the top 1,000 ranked for model A identical or in high LD (*r*^2^ > 0.90) with a SNP among the top 1,000 ranked from model B. This approach may yield different results depending on whether starting the comparison from model A to model B or vice versa and, therefore, here we report results for both directions.

## Results

### Model performance

**Fig. 2.**
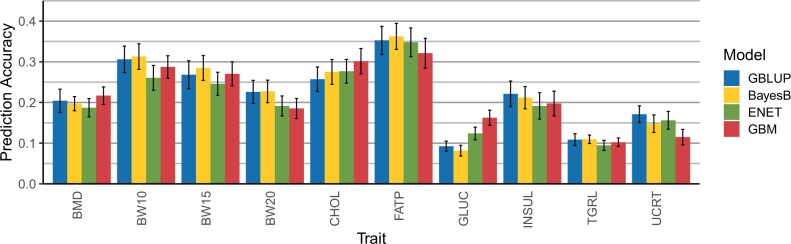
Prediction accuracy, including standard errors, obtained from GBLUP, BayesB, ENET, and GBM for the traits: BMD, BW10, BW15, and BW20, CHOL, FATP, GLUC, TRGL, INSUL, and UCRT.

The accuracy of predicted phenotypes from GBLUP, BayesB, ENET, and GBM for animals in the validation set (generation 11) is shown in Fig. 2. The best performing model varied according to the trait being analyzed.

Prediction accuracies obtained for traditional linear models (GBLUP and BayesB) were, in general, proportional to the trait’s heritability, with GBLUP overcoming BayesB for BMD, GLUC, INSUL, TRGL, and UCRT. Predictive accuracy obtained with GBLUP was never the worst among tested models for any of the traits. The highest prediction accuracies were observed for body composition traits (BW10, BW15, BW20, and FATP), for which BayesB outperformed all other models. Conversely, BayesB particularly underperformed when analyzing GLUC which was one of the traits with the lowest overall accuracy across linear models. The ENET had lower prediction accuracy when compared to other models across traits. It was never the best performing model for a particular trait and showed the worst performance for BMD, BW10, BW15, BW20, INSUL, and TRGL.

The GBM model showed best predictive performance for BMD, CHOL, and GLUC. For other traits, prediction accuracy from GBM varied from being competitive to the linear models for BW10, BW15, and TRGL, to a poorer performance observed for UCRT. It only showed the worst predictive ability among all models for FATP, but with a small difference from the next performing model. The GBM model performed particularly well when analyzing GLUC, showing predictive performance much higher than the linear models. Overall, GBM showed a less consistent pattern of predictive performance across trait categories when compared to the linear models.

In terms of prediction error, GBLUP was the model with best performance for most traits, in most cases followed by GBM (Table 2). The GBM model showed the lowest RRMSE for BMD, CHOL, and GLUC. For all traits, BayesB showed the highest RRMSE when compared to other models, even for traits for which it had the best prediction accuracy. Relative differences between RRMSE from the best and worst model were lower for body weight traits (BW10, BW15, and BW20) and higher for CHOL and INSUL.

**Table 2.** RRMSE obtained from GBLUP, BayesB, ENET, and GBM for 10 phenotypes analyzed in the diversity outbred mouse population.

Trait	GBLUP	BayesB	ENET	GBM
BMD	0.94	0.97	0.95	**0.93**
BW10	**0.72**	0.81	0.76	0.75
BW15	**0.71**	0.76	0.73	0.72
BW20	**0.71**	0.75	0.72	0.74
CHOL	0.80	0.94	0.86	**0.78**
FATP	**0.71**	0.74	0.72	0.72
GLUC	0.94	0.99	0.95	**0.91**
TRGL	**1.02**	1.18	1.09	1.06
INSUL	**0.83**	0.88	0.86	0.84
UCRT	**0.88**	0.95	0.91	0.90

Per trait, values represent standard deviations from the phenotypic mean. The lowest value for each trait is indicated in bold.

### Impact of feature selection on prediction performance

**Fig. 3.**
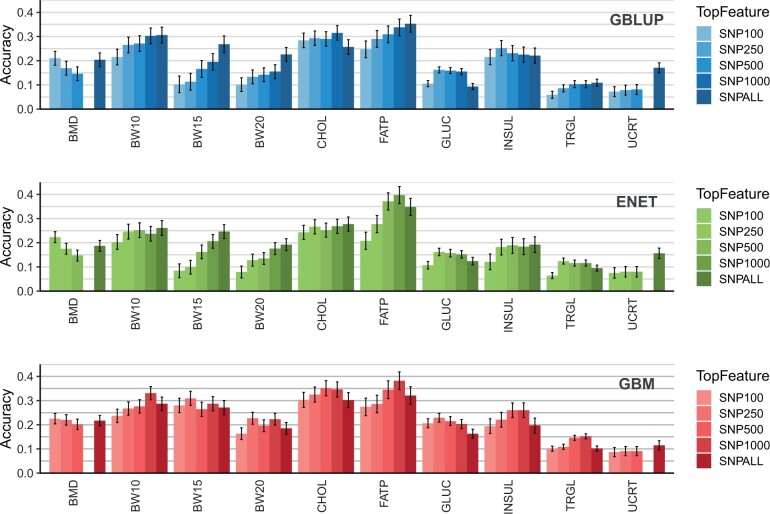
Prediction accuracy, including standard errors, for the analyzed traits for GBLUP (top), ENET (mid), and GBM (bottom) fitting exclusively the top 100 (SNP100), 250 (SNP250), 500 (SNP500), and 1,000 (SNP1000) ranked by a GBM model and fitting all SNPs (SNPALL). Traits: BMD, BW10, BW15, and BW20, circulating cholesterol at 19 weeks (CHOL), FATP, GLUC, TRGL, INSUL, and UCRT.

Figure 3 shows the prediction accuracy obtained by GBLUP, ENET, and GBM when fitting only the 100, 250, 500, and 1,000 SNP selected as most important features from a GBM model for all SNPs (52K). Results for prediction error (RRMSE) are presented in Supplementary Fig. 2. When compared to fitting all SNPs (SNPALL), fitting only a subset of important features showed distinct pattern depending on the trait analyzed and model applied.

When fitting the GBLUP model, including increasingly more important SNPs resulted, for most traits, in an incremental increase in accuracy, reaching its maximum value in the SNPALL scenario. This was especially the case for traits, which were expected to be highly polygenic like BW10, BW15, BW20 and FATP. For CHOL, GLUC, and INSUL, fitting GBLUP with a subset of top importance SNPs selected by the GBM model yielded higher accuracy than SNPALL, the number of top SNPs that resulted in the highest prediction accuracy was dependent on the trait being analyzed.

When fitting ENET, including subsets of relevant SNP as predictors for BW10, BW15, and BW20 yielded similar results as for GBLUP with the accuracy increasing with the inclusion of more markers. For FATP, there was an incremental increase in accuracy by including more important SNPs, but accuracy from SNP500 and SNP1000 was even higher than from SNPALL and comparatively higher than the accuracies obtained for FATP by GBLUP. The pattern observed for accuracies obtained when fitting different number of preselected markers in the ENET model was less linear for the other traits.

The GBM model showed for almost all traits a higher predictive accuracy when considering a subset of SNPs compared to fitting all available SNP (SNPALL). The only exception to that was UCRT, for which the inclusion of important SNPs up to 500 resulted in only a marginal increase in accuracy. For each tested subset of important SNPs, GBM outperformed GBLUP and ENET for prediction accuracy, except for FATP. For this trait, ENET yielded around 0.02 higher absolute accuracy than GBM for SNP1000. For BMD and UCRT, the total number of features selected by GBM was 364 and 419. Consequently, for these traits, running SNP1000 was not possible and SNP500 indicates SNP364 and SNP419.

### Generation gaps and connectedness between reference and validation sets

**Fig. 4.**
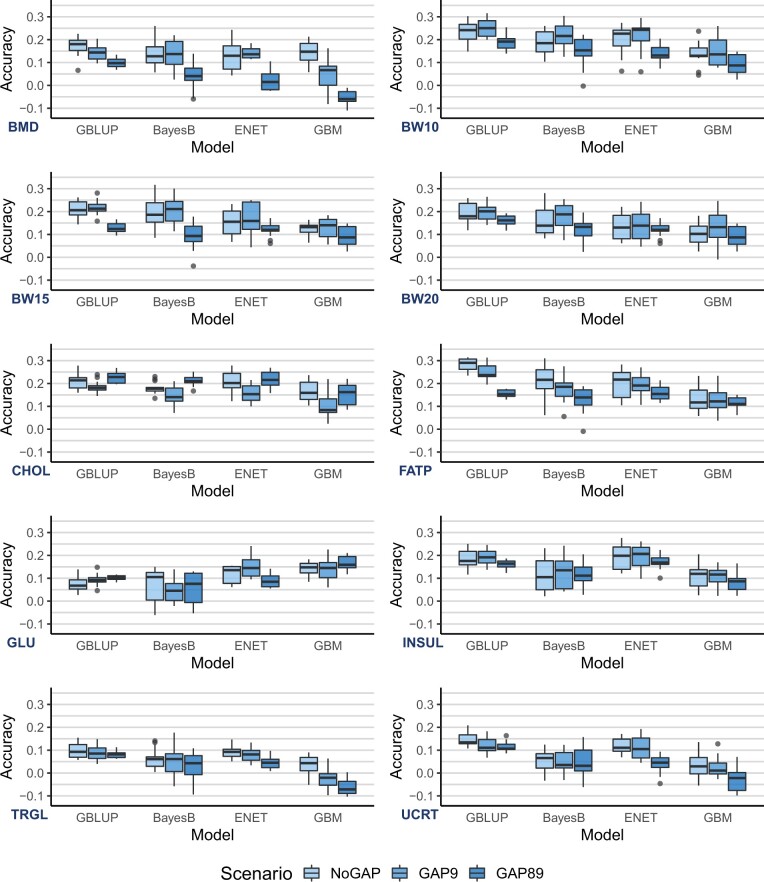
Distribution of prediction accuracies (from 20 replicates) for scenarios including progressive distance between reference and validation sets using GBLUP, BayesB, ENET and GBM models. Traits: BMD, BW10, BW15, and BW20, CHOL, FATP, GLUC, TRGL, INSUL, and UCRT.

Prediction accuracies obtained for scenarios with increasing distance between reference and validation sets decreased for almost all trait/model combinations, in different magnitudes (Fig. 4). An exception to this pattern was observed for GLUC, which showed a marginal increase in accuracy (although not significantly different across scenarios) for GBLUP and GBM. Independent of the trait analyzed or model used, differences in accuracy between NoGAP and GAP9 were much lower than between NoGAP and GAP89 or between GAP9 and GAP89. These differences varied from −0.20 (BMD—GBM) to +0.03 (GLUC—GBLUP). Results for prediction error (RRMSE) on these same scenarios are presented in Supplementary Fig. 3. Overall, both accuracy and prediction error showed a similar pattern across traits and models.

The GBLUP model showed the lowest decrease in accuracy between NoGAP and GAP89 scenarios among traits when compared to other models, except for FATP, for which the difference in performance between NoGAP and GAP89 for GBLUP was the highest among all models (−0.12). On the other hand, the GBM model showed the highest drop in accuracy when comparing NoGAP and GAP89 scenario, especially for BMD, TRGL, and UCRT. Especially for these traits, using GBM on a GAP89 scenario resulted in negative average prediction accuracies.

Independent of the model used, the traits BW10, BW15, BW20, and FATP showed the lowest decrease in accuracy while BMD, TRGL, and UCRT showed the highest decrease in accuracy between NoGAP and GAP89 scenarios. For CHOL, the prediction accuracy of GAP89 was higher than observed for GAP9 for all models tested, while for GLUC this pattern was observed for predictions from GBLUP, BayesB, and GBM, although in smaller differences between scenarios.

The ranking of model accuracy across traits observed using the full dataset (Fig. 2) and for the generation gap scenarios (Fig. 4) was not the same. When considering the full dataset, GBM yielded the best accuracy for BMD, CHOL, and GLUC; however, the same pattern was not observed for the generation gap scenarios. Overall, for any of the generation gap scenarios, GBLUP had the best accuracy across traits.

### Animal predictions and SNP ranking similarities between models

**Fig. 5.**
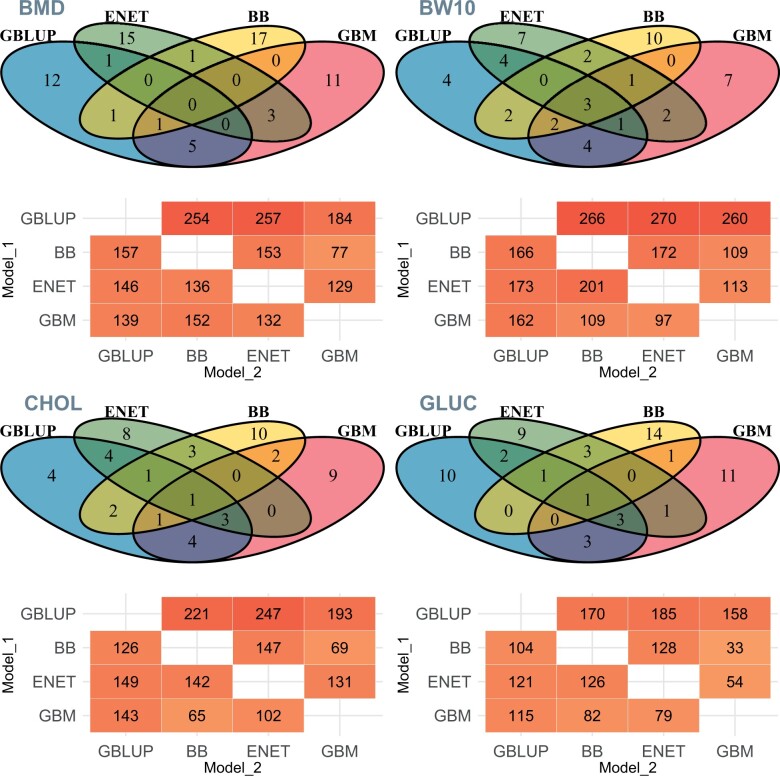
Venn diagrams showing the unique animals among the top 20 (above) predicted values (10% of the validation subset) between models and the number of SNP markers in common or in high LD (*r*^2^ > 0.90) among the top 1,000 SNP (below) from GBLUP, BayesB (BB), ENET, and GBM for BMD, BW10, CHOL, and GLUC. Values represent the overlap of SNP when Model_1 (*y*-axis) is considered as reference. Traits: BMD, BW10, CHOL, and GLUC.

The number of unique animals among the top 20 ranked for BMD (Fig. 5, top left), BW10 (Fig. 5, top right), CHOL (Fig. 5, bottom left), and GLUC (Fig. 5, bottom right) was 12, 4, 4, and 10 for GBLUP; 17, 10, 10, and 14 for BayesB; 15, 7, 8, and 9 for ENET; and 11, 7, 9, and 11 for GBM, respectively. The number of animals uniquely in common between any model and GBM varied between 0 and 5 for BMD, 0 and 5 for BW10, 0 and 4 for CHOL, and 0 and 3 for GLUC. Overall, the number of overlapping animals between pairs and triples of models was slightly higher for BW10 than for the other 3 traits (BMD, CHOL, and GLUC). Results for all traits are included in Supplementary Fig. 4.

The count of overlapping markers among the top 1,000 ranked across models investigated was higher for BW10 than for the other 3 traits known to be partially under epistatic control (Fig. 5, below within traits), with GLUC showing the lowest overlapping overall. Higher values were usually observed for comparisons between 2 linear models than between a linear model and GBM. When comparing model pairs, the lowest overlap was observed between ENET and GBM and between BayesB and GBM. Comparisons between GBLUP and any other model had more overlapping markers than between the other models. The largest differences between values above diagonal and the respective comparison below diagonal were observed for comparisons between GBLUP and any other model, with values above the diagonal (GBLUP × other model) being considerably higher than values below the diagonal (other model × GBLUP).

## Discussion

In the present study, we compared predictive performances of commonly applied linear methods (GBLUP, BayesB, and ENET) and a nonparametric ML ensemble method (GBM) for GP of 10 complex phenotypes in the DO mouse population. Although the evaluation of routine implementation of genomic selection in mice was not our focus, results of predictive accuracy can be used as a guide if selection is intended for this population. Currently, the mating scheme used for the DO population is a randomized outbreeding strategy (Churchill et al. 2012); however, being able to predict phenotypes could be useful if any directional selection is of interest in the future.

Accuracies of GP have been reported by previous authors in another mice population (Lee et al. 2008; Legarra et al. 2008). Overall results showed low-to-medium predictive accuracies, ranging from 0.10 to 0.65 depending on the trait analyzed and cross-validation strategy considered. Our results confirmed that the performance of GP methods seems to be highly dependent on the trait’s genetic architecture. When analyzing the traits that are mostly polygenic (BW10, BW15, BW20, FATP, and TRGL), linear models were able to outperform GBM in both the full dataset (Fig. 1) and for scenarios with lower connectedness between reference and validation subsets (Fig. 4). BayesB was the best model for the 3 BW traits and FATP, while GBLUP had the best results for INSUL, TRGL, and UCRT. In a previous study, Zhang et al. (2012) showed an absence of QTL with pronounced effects for this TRGL, with mostly small effects detected for genetic markers. This could explain why GBLUP had better predictive performance than BayesB or ENET for this trait.

Among the 10 traits analyzed, evidence of nonadditive effects has been reported for BMD (Tyler et al. 2016), CHOL (Li and Churchill 2010; Stewart et al. 2010), and GLUC (Stewart et al. 2010; Chen et al. 2017). We also found suggestive results when estimating the variance from additive-by-additive effects using the present dataset (Supplementary Table 1). Coincidently for these traits, GBM showed a better predictive performance than the linear models in the full dataset. In a detailed simulation study, Abdollahi-Arpanahi et al. (2020) showed that for traits controlled by many QTL (1,000) with only additive effects, GBLUP and BayesB outperformed any ML approach, while for traits controlled by a small number of QTL (100) with nonadditive effects, GBM largely outperformed other parametric and nonparametric models.

Results on the performance of GP using real data have shown more inconsistent results. Azodi et al. (2019) have performed a detailed benchmarking of parametric and nonparametric models for GP in plants and reported no clear association between prediction performance of ML models to the genomic architecture of the traits. While exploring GP on a large human dataset, Bellot et al. (2018) have also observed that ML algorithms did not necessarily outperform linear models even when the genetic variance coming from dominance effects was around 50% of the variance from additive effects. On the other hand, Zingaretti et al. (2020) investigated the performance of GP in strawberry using CNN and reported that ML methods may outperform parametric and semiparametric models when the epistatic component is relevant (proportionally to the additive genetic variance) and narrow-sense heritability is medium to low (below 0.35). This is roughly in line with our results for CHOL (*h*^2^ = 0.29), GLUC (*h*^2^ = 0.18), and BMD (*h*^2^ = 0.36). Interestingly, in our results, the superiority of predictive ability from GBM compared to the parametric models was higher for the trait with lower heritability (GLUC) than for CHOL and BMD. Low-heritability traits imply that a smaller portion of observed variance is explained by the additive component, and therefore, any other nonadditive effects might explain proportionally more of the phenotypic variance than in high-heritability traits. This larger proportion of the phenotypic variance with a nonadditive origin can more easily be captured by the GBM model, increasing performance of the model for such traits. One similarity between the present study and Zingaretti et al. (2020) is that both worked with outbred populations, which imply a higher level of heterozygous genotypes than usually found in elite (purebred) individuals. This increased frequency of heterozygous loci may allow ML models to capture nonadditive effects more easily and, therefore, outperform linear models in prediction performance when such effects are relevant. Thus, the similarity between results obtained in the present and afore-mentioned studies is in line with the current knowledge of genetic architecture of the analyzed traits (Table 1).

The efficient built-in feature extraction from GBM enables prescreening of SNPs (Lubke et al. 2013; Li et al. 2018) and, therefore, minimizes the loss in accuracy when reducing the number of markers in a genotype panel. The performance of GBM on preselection of informative SNP markers varied across traits and models subsequently used for phenotype prediction. When considering the highly polygenic traits (BW10, BW15, BW20, FATP, and TRGL), using preselected SNP markers generally decreased the accuracy of GBLUP. However, for ENET and GBM, in certain situations, a subset of preselected SNP tended to yield higher predictive accuracy than using the complete SNP panel. For traits with evidence of nonadditive effects (BMD, CHOL, and GLUC), a similar pattern was observed, with the difference that the use of subsets of markers more commonly resulted in higher predictive accuracy than when fitting the models with all available SNP. After preselection of informative markers, GBM showed the biggest gains in accuracy across traits and models, which is expected, since we used a GBM model to accomplish the former. Azodi et al. (2019) observed that feature selection (using the random forest method) notably improved prediction accuracies when using artificial neural networks (ANN) in multiple plant species. However, in their case, predictive accuracies using ANN were overall lower than other models. Using data from Brahman cattle, Li et al. (2018) investigated the potential of 3 different ensemble learning methods to preselect SNPs and showed that GBLUP accuracies using SNPs preselected with GBM in some cases were actually similar to accuracies based on all SNPs. Together with our findings, the above-mentioned results suggest that GBM can be used for prescreening informative markers, even when further GP is performed using traditional linear models, such as GBLUP. One limitation of ours and all investigations found in literature is the focus in performing feature selection and further fitting top relevant markers into univariate models. Further research is needed to expand this from a univariate to multivariate approach for practical implementation in genomic selection breeding programs.

Curiously, for UCRT (and partly for BMD), the inclusion of preselected SNP (from 100 to 500) did not increase predictive accuracy, which was similar across scenarios and models, but always lower than using the full SNP panel. To understand this pattern, it is important to remember that these SNP were extracted as important features from a previous GBM model. This process is completely dependent on reference data and may be affected by nonadditive effects captured by the GBM model. In such a reduced subset (100–1,000 from a total of 50,112), GBM could be choosing SNP as relevant while these are placed in redundant regions of the genome or that are involved in relevant epistatic events. In both cases, the inclusion of such SNPs does not include relevant information for linear models, resulting no positive impact in predictive accuracies. A similar pattern was previously reported by Azodi et al. (2019) when fitting different numbers of informative preselected markers into a model for GP in sorghum. Authors observed low and stable prediction accuracy (around 0.40) when using up to 5% of top markers, but a strong increase when using more than 5% of top relevant markers, reaching up to 0.60 when using 80% of available markers. We have replicated the feature selection of top 100, 250, 500, and 1,000 SNPs using BayesB instead of GBM and results suggest a superiority of GBM for preselecting informative markers (Supplementary Fig. 5) as predictive accuracy across traits was consistently lower when using BayesB compared to using GBM for the same task.

The size of the reference population and the strength of the connectedness between reference and validation subsets have been shown to influence GP accuracies from linear models (Habier et al. 2007; Wientjes et al. 2013; Liu et al. 2015). In terms of connectedness, maximizing predictive performance involves maximizing connectedness between reference and validation populations, while simultaneously minimizing connectedness within the reference population (Pszczola et al. 2012). Although extensive research has been done over this topic regarding traditional GP using parametric models, this is not the case for ML models.

There is quite a difference in size when comparing the DO mouse data to datasets commonly used in the context of animal breeding, which are usually around thousands and not rarely at millions of observations. In addition to that, much has been discussed in literature about how “data-hungry” ML models could be (Xiong et al. 2015; Montesinos-López et al. 2019). However, studies have not only shown no clear superiority of predictive performance from ML over parametric models when using large datasets (Bellot et al. 2018), but also good performance of the same ML models when using datasets of hundreds or few thousands of individuals (Azodi et al. 2019; Zingaretti et al. 2020; Bargelloni et al. 2021). Although the number of observations (phenotypes and genotypes) available for the present study was limited and, therefore, results reported should be interpreted with caution, we believe that the deeper knowledge on genetic architecture of traits and the already discussed structure of the DO Mouse population strengthen the evidence of findings presented in this study.

In this study, when compared to the predictive performance of linear models, GBM had competitive results for most traits and a superior performance for BMD, CHOL, and GLUC when using the full dataset (Fig. 2). However, this relatively good performance was not maintained for NoGAP, GAP9, and GAP89 scenarios that contained less data (Fig. 4). This pattern was observed across all traits and scenarios and may indicate that using only 300 individuals in the reference subset affected more drastically the predictive performance of the GBM model than GBLUP, BayesB, or ENET. Overall, the decrease in accuracy observed from NoGAP to GAP89 was also more severe for GBM than for other models. We hypothesize that this could happen because as the distance between reference and validation populations increases, the frequency of recombination events also increases between genotypes from individuals in the 2 subsets. As GBM implicitly fits SNP × SNP interactions, the increased number of recombinations will impair the accurate estimation of allele combinations and interactions.

The aim of GP in the breeding context is to make accurate selection decisions early in the animal’s life. Therefore, comparing the top ranked individuals between methods is a useful way to understand how different these are in practical terms. In the present study, independent of the trait analyzed, linear models shared many more individuals among the top 20 best from the 3 models (GBLUP, BayesB, and ENET) than with GBM. For GLUC, for which we expected nonadditive effects, the similarity between rankings for linear models was lower, while the number of unique animals for a single model was higher. On the other hand, as we consider BW10 to be controlled mostly by additive effects, the absence of relevant nonadditive effects is probably the cause of lesser differences between linear models and GBM regarding selection decisions.

We evaluated the overlap among top ranked SNP between the different models (Fig. 5 and Supplementary Fig. 6). One thing that must be acknowledged is that there are differences in the way each of the different models estimate the relevance of a single SNP. This may affect the comparison of the overlapping relevant genomic regions between methods for a certain trait. For the linear models, SNP relevance is based on changes observed at the phenotypic level by the change in allelic dosage (0, 1, 2), while for GBM an SNP is considered relevant when the inclusion of this SNP in the decision tree contributes to a reduction in prediction error, and this can be affected by another SNP also used in the same decision tree. On the other hand, when used for GP, these differences will impact the obtained results and thereby indirectly impact selection decisions. Therefore, this simple comparison of SNP ranks is informative to understand the similarity of outcomes from different models.

The asymmetry of results obtained from the overlapping top ranked SNP between models can be seen comparing values below and above diagonals in Fig. 5. The strongest driver of the differences observed seems to be the ability of models to perform variable selection. When starting comparisons from GBLUP (first row above diagonals in Fig. 5), there were many SNPs located in specific short genomic regions among the top 1,000 ranked SNP for this model. Several top markers from GBLUP were in high LD with at least 1 top ranked marker from the other models. In contrast, the variable selection applied by BayesB, ENET and GBM, resulted in fewer SNPs within a given genomic region to be among the top ranked ones. Consequently, the number of top ranked SNP in high LD with top ranked SNPs from the other models was much lower. Therefore, the difference between values above and below diagonal is directly related to the difference in magnitude of penalization applied to markers between any given pair of models. When comparing results from GP of height in maize using BayesA, ENET, and random forest models, Azodi et al. (2019) have observed marked dissimilarity among the top 8,000 markers. Results showed that BayesA and ENET shared 1,589 (20%) markers, while RF shared 328 (4%) markers with BayesA and 475 (6%) with ENET. In the present study, this higher similarity among SNP ranks between linear models in addition to much lower similarity between linear models and an ensemble ML model [random forest in Azodi et al. (2019) or GBM in the present study] was also observed for BW10. At the same time, the difference between average SNP overlaps between 2 linear models or between a linear model and GBM was much lower for GLUC, which coincidently was the trait with strong suggestion of relevant portion of variance coming from the epistatic component (Supplementary Table 1). One important aspect to note is that although marked differences were observed between polygenic and epistatic traits in terms of SNP overlaps, there was no one clear pattern observed across the epistatic traits. This might have occurred because although these 3 traits (BMD, CHOL, and GLUC) are affected by epistatic effects, genetic architecture is not the same between them in terms of magnitude of epistatic effects and position of such epistatic QTL on the genome. From these results, we can hypothesize that linear models have similar SNP rankings for polygenic traits because the underlying genetic architecture is in line with assumptions and parametrization considered in such models, while the presence of nonadditive effects is probably captured differently by the distinct linear models, generating the observed overall dissimilarity.

## Conclusions

GBM had a competitive performance for GP of complex phenotypes in mouse specifically for traits with nonadditive effects where it can outperform linear models. The GBM was more affected by datasets with less data points and by decrease in relationship between reference and validation populations than linear models. Considerable differences between the top ranked animals suggest that using linear models vs GBM will result in clear differences in selection decisions. The built-in feature selection from GBM seems beneficial to extract a smaller number of informative markers and in some cases can improve accuracies even when parametric models are used for prediction.

## Data availability

All data associated with this manuscript, and the code developed and used to perform analyzes described in this manuscript, can be obtained at https://doi.org/10.6084/m9.figshare.15081636.v1. All software used is publicly available. The Supplemental Material includes a detailed description of results.

Supplemental material is available at *G3* online.

## Funding

This study is part of the GENE-SWitCH project that received funding from the European Union’s Horizon 2020 research and innovation programme under grant agreement no. 817998. Gary Churchill acknowledges support by the National Institutes of Health (NIH) grant R01 GM070683. 

## Conflicts of interest

The authors report no conflicts of interest related to the present manuscript. 
